# In Vitro Assessment of the Synergistic Effect of Aspirin and 5-Fluorouracil in Colorectal Adenocarcinoma Cells

**DOI:** 10.3390/curroncol30070460

**Published:** 2023-06-27

**Authors:** Monica Susan, Ioana Macasoi, Iulia Pinzaru, Cristina Dehelean, Iosif Ilia, Razvan Susan, Ioana Ionita

**Affiliations:** 1 Faculty of Medicine, “Victor Babeș” University of Medicine and Pharmacy from Timisoara, Eftimie Murgu Square No. 2, 300041 Timisoara, Romania; 2 Faculty of Pharmacy, “Victor Babes” University of Medicine and Pharmacy from Timisoara, Eftimie Murgu Square No. 2, 300041 Timisoara, Romania; 3 Research Center for Pharmaco-Toxicological Evaluations, Faculty of Pharmacy, “Victor Babes” University of Medicine and Pharmacy from Timisoara, Eftimie Murgu Square No. 2, 300041 Timisoara, Romania

**Keywords:** 5-fluorouracil, aspirin, bcl-2 proteins, colorectal adenocarcinoma, caspases, immunofluorescence staining

## Abstract

Although remarkable progress has been made, colorectal cancer remains a significant global health issue. One of the most challenging aspects of cancer treatment is the resistance of tumor cells to classical chemotherapy. Conventional therapy for colorectal cancer often involves the use of 5-fluorouracil as a chemotherapeutic agent. Aspirin, a drug used primarily to prevent cardiovascular complications, became a focus of attention due to its potential use as an antitumor agent. The purpose of the study was to evaluate the potential synergistic cytotoxic effects of aspirin and 5-fluorouracil on colorectal adenocarcinoma cells. The viability of cells, the impact on the morphology and nuclei of cells, the potential antimigratory effect, and the impact on the expression of the major genes associated with cell apoptosis (Bcl-2, Bax, Bad), as well as caspases 3 and 8, were evaluated. The results indicated that the two compounds exerted a synergistic effect, causing a reduction in cell viability accompanied by changes characteristic of the apoptosis process—the condensation of nuclei and the reorganization of actin filaments in cells, the reduction in the expression of the Bcl-2 gene, and the increase in the expression of Bax and Bad genes, along with caspases 3 and 8. Considering all these findings, it appears that aspirin may be investigated in depth in order to be used in conjunction with 5-fluorouracil to increase antitumor activity.

## 1. Introduction

Colorectal cancer (CRC) refers to a group of neoplasms that include colon cancer and rectal cancer. It is a significant global health problem, since it is the third most frequently diagnosed and second most lethal type of cancer in the world [1]. Further, epidemiological studies have highlighted that in 2020, approximately 10% of cancer-related deaths were attributable to CRC. The prediction that CRC cases will double by 2035, particularly in developing countries, is alarming. Associated with the abnormal proliferation of glandular epithelial cells in the colon, CRC exclusively affects the colon and rectum [2]. There is no clear explanation of the etiology of colorectal cancer as of today; however, numerous risk factors have been studied, such as age over 50, environmental and lifestyle factors, high-fat diets, low calcium and folic acid intake, and others [3]. Additionally, genetic factors were discussed. In the elderly, long-term ulcerative colitis and Crohn’s disease are closely associated with the risk of developing CRC [4].

It has been shown that CRC is closely linked to several other pathologies, such as adenomatous polyposis syndromes, acromegaly, diabetes mellitus, cardiovascular disease, and others [5,6,7]. A number of emerging studies suggest that coronary heart disease and colorectal cancer may be interrelated, in part due to the presence of common risk factors such as obesity, sedentary lifestyles, or smoking [8,9,10]. Conversely, patients with colorectal cancer have a higher risk of developing cardiovascular diseases, including myocardial infarction and stroke, which were twice as likely as in a non-cancer control group [11]. Patients who undergo surgical resection also face this risk of myocardial infarction in the short term [12]. Moreover, CRC patients have other cardiovascular risk factors, such as chronic inflammation caused by cancer and antitumor therapies that contain antimetabolites, such as 5-fluorouracil [13]. Statistically, cardiovascular diseases caused by complications of antitumor therapy rank among the leading causes of death [14,15]. Therapy for CRC, despite its remarkable progress, is limited by the non-specific targeting of tumor cells and systemic adverse reactions [16]. 

Chemotherapy, radiotherapy, and surgical intervention are the main therapeutic options for patients diagnosed with CRC [17]. Currently, antitumor chemotherapy consists of single agents, primarily fluoropyrimidine, such as 5-fluorouracil (5-FU), or combinations of two or more antitumor agents. Although the combination of several chemotherapeutic compounds can be beneficial from a therapeutic standpoint, it is often accompanied by serious, irreversible adverse reactions. As a result, treatment with a single chemotherapeutic agent is preferred when patients are in the early stages of the disease, with a low risk of deterioration [2]. The clinical benefits of 5-FU, a synthetic fluorinated pyrimidine analog, are, however, typically short-lived, and the treatment does not completely eradicate tumor cells. Therefore, the recurrence rate following 5-FU treatment is relatively high [18]. Furthermore, one of the major reasons why 5-FU treatment fails to produce the desired results is because of the development of resistance to the treatment, particularly in patients with metastatic cancer. In managing metastatic disease over the long term, this is one of the most challenging aspects [19]. Further, 5-fluorouracil resistance was linked to PI3K/Akt signaling in cancer stem cells [20]. In addition, 5-FU increases the production of reactive oxygen species in tumor cells, which is one of its main mechanisms of action. There is evidence that cancer stem cells are able to develop protective mechanisms against reactive oxygen species, making them resistant to treatment with 5-FU [21].

As threats to the prognoses of patients, cancer stem cells can cause resistance to therapy and encourage metastasis. The eradication of cancer stem cells can therefore be an important strategy in the treatment of colon cancer [22]. This has led to a current medical practice that involves the combination of 5-FU with other compounds to reduce side effects, overcome clinical resistance, and enhance 5-FU’s antitumor properties [23]. A large number of drugs that are already approved for use as therapy for other pathologies were tested for their antitumor effects, as the process of developing new drugs is costly and time-consuming, and most of the drugs that passed through the preclinical phase did not succeed in clinical trials. This resulted in the concept of “old medicine, new tricks” [24]. 

At the end of the 1800s, acetylsalicylic acid (aspirin—ASA) was discovered and used as an analgesic. Upon its introduction into therapy, aspirin was proved to have new therapeutic properties, so it is currently used for its anti-inflammatory properties and for the prevention of arterial thrombosis and venous thrombosis [25]. There is evidence to suggest that the long-term use of aspirin can decrease the risk of cardiovascular disease, stroke, and gastrointestinal cancer [26,27,28]. According to recent research, aspirin may prevent the development of other types of cancer, including colorectal cancer [29]. The use of aspirin, due to its non-selective inhibiting effect on cyclooxygenase (COX), has the effect of suppressing inflammation and inhibiting the development of cancer [30]. Although aspirin inhibits COX, other mechanisms are also associated with its potential antitumor effects, including the inhibition of cyclin-dependent kinases (CDKs) by ASA metabolites, the activation of AMP-kinase, and the inhibition of mTOR signaling, or decreases in gene expression that may have an impact on tumor development [31]. Additionally, ASA inhibits the growth of colorectal cancer stem cells by interfering with the Wnt/β-catenin signal pathway [32]. Moreover, prostaglandin E2 (PGE2) is well known to encourage the proliferation of normal and tumor stem cells [33]. Consequently, aspirin can reduce the proliferation of cancer stem cells by inhibiting the production of PGE2 [34]. This is of particular importance, since after chemotherapy, PGE2 levels increase, stimulating tumor stem cells to divide, thereby repopulating the tumor [35]. Studies have also indicated that ASA inhibits the expression of stemness genes, which play a role in inflammation. By mediating histone methylation, ASA inhibits the transcription of pro-inflammatory stemness genes. It is possible that these mechanisms contribute to the possible inhibition of cancer stem cells by aspirin and the prevention of the progression of cancer [36]

Recently, it was demonstrated that the use of aspirin can improve clinical results for patients with metastatic CRC who are treated with a 5-FU derivative [37]. This study raised the hypothesis that aspirin may enhance the chemosensitivity of CRC cells to basic chemotherapy, such as 5-FU. Nevertheless, the mechanisms underlying aspirin’s antitumor effect are complex and remain incompletely understood.

As a result of these premises, the purpose of the present study was to investigate the antitumor potential of ASA, but also of the combination of ASA and 5-FU at the level of colorectal adenocarcinoma cells—HT-29. The cytotoxic effect was evaluated by examining the cell viability, cell morphology, immunofluorescence, and cell migration, as well as by measuring the gene expression and caspases associated with the apoptosis process.

## 2. Materials and Methods

### 2.1. Reagents

The analyzed compounds (aspirin and 5-fluorouracil) and the other reagents used in the present study dimethyl sulfoxide (DMSO), fetal calf serum (FCS), penicillin/streptomycin, trypsin-EDTA solution, phosphate saline buffer (PBS), MTT (3-(4,5-dimethylthiazol-2-yl)-2,5-diphenyltetrazolium bromide), 4′,6-Diamidino-2-phenylindole dihydrochloride, and 2-(4-Amidinophenyl)-6-indolecarbamidine dihydrochloride (DAPI), were purchased from Sigma Aldrich, Merck KgaA (Darmstadt, Germany), and Alexa Fluor^®^ 555 Phalloidin was acquired from Cell Signaling USA.

Cell lines were cultured in the specific media DMEM (P04-03550) and McCoy’s 5A (P04-05500), which were purchased from PAN Biotech GmbH (Aidenbach, Germany). As part of RT-PCR, the following primers were used: 18S, Bax, Bcl-2 purchased from Thermo Fisher Scientific, Inc. (Waltham, MA, USA) and Bad, caspase 3, and caspase 8, purchased from Eurogentec (Seraing, Belgium). All reagents presented appropriate characteristics for use in cell culture. 

### 2.2. Cell Culture

The cytotoxic potential of samples (ASA, 5-FU, and the combination of ASA and 5-FU) was evaluated using the healthy human keratinocyte cell line, HaCaT (catalog number: 300493), purchased from CLS Cell Lines Service GmbH in frozen vials, and the colorectal adenocarcinoma tumor cell line, HT-29 (catalog number: HTB-38^TM^), which was provided by ATCC (American Type Cell Collection, Lomianki, Poland), in frozen vials. The cells were cultured in specific culture medium as follows: HaCaT in DMEM medium, and HT-29 in McCoy’s 5A culture medium, both media supplemented with 10% FCS and penicillin/streptomycin mixture. During the experiment, the cells were maintained according to the recommendations (37 °C and 5% CO_2_).

### 2.3. Cellular-Viability Assessment

To assess the impact of the analyzed compounds at the level of cell viability, the MTT method was applied. In order to conduct this determination, 96-well plates were used for cell cultivation, with stimulation occurring at a suitable confluency level with ASA (1, 2.5, 5, 7.5 and 10 mM), 5-FU (5, 10, 25, 50 and 75 µM), and the combination of ASA (2.5 mM) and 5-FU (25 μM) for an interval of 72 h. After this time interval, the culture medium was changed with 100 μL/well of fresh medium, and MTT reagent was added (10 μL/well) for a period of 3 h. At the end of the procedure, a solubilization solution (100 μL/well) was added and allowed to stand for 30 min. A 570-nm-wavelength absorbance measurement was conducted to calculate cell viability using a Cytation 5 device (BioTek Instruments Inc., Winooski, VT, USA).

### 2.4. Cellular Morphology

The cell morphologies of HaCaT and HT-29 cells were analyzed after 72 h of stimulation to assess the compounds’ cytotoxic potential. 

To assess the impact on the morphologies of the cells, they were photographed under brightfield lighting conditions using an Olympus IX73 inverted microscope (Olympus, Tokyo, Japan). The cellSens Dimensions v.1.8 software package (Olympus, Tokyo, Japan) was used to process the images for analysis.

### 2.5. Immunofluorescence

The effects induced by the samples at the level of nuclei and actin filaments were examined by immunofluorescence studies. Thus, the cells were cultured in 12-well plates and stimulated with ASA 2.5 mM, 5-FU 25 μM, and the combination of these, at the time interval previously used to determine cell viability.

Ice-cold PBS was used to wash the cells, and 4% paraformaldehyde was used for fixing. Actin filaments were visualized using Rhodamine Phalloidin, whereas nuclei were visualized using DAPI, after Triton X 0.2% permeabilization. Photographs were taken using an Olympus IX73 inverted microscope with a DP74 camera, and images were processed and analyzed with the CellSens software, version 1.18. The apoptosis index (AI) parameter was employed for quantitative determination of the apoptosis process [38]:

$$\mathrm{Apoptotic}\;\mathrm{index}\;(\mathrm{AI})\;(\%)=\frac{\mathrm{Number}\;\mathrm{of}\;\mathrm{apoptotic}\;\mathrm{cells}}{\mathrm{Total}\;\mathrm{number}\;\mathrm{of}\;\mathrm{cells}}\;\times \;100$$

### 2.6. Wound-Healing Assay

In order to determine how the compounds affected cell migration, the wound-healing (scratch) method was used. Cells were cultured in 24-well Corning plates, and after reaching the appropriate confluence, the AutoScratchTM Wound Making Tool (BioTek^®^ Instruments Inc., Winooski, VT, USA) was used according to the manufacturer’s recommendations. Following the photography of the cells (T0), they were stimulated for 24 h with ASA 2.5 mM, 5-FU 25 μM, and ASA 2.5 mM + 5-FU 25 μM. After this time interval, the cells were photographed again (T24) and migration rate was calculated using the formula previously described [39]. Cells were photographed using Cytation 1 and processed using the dedicated software.

### 2.7. Quantitative Real-Time Polymerase Chain Reaction (qRT-PCR)

The PeqGold RNAPure^TM^ Package was applied to extract RNA according to the protocol recommended by the manufacturer (Peqlab Biotechnology GmbH, Erlangen, Germany) and the amount obtained was determined using a DS-11 spectrophotometer (DeNovix, Wilmington, DE, USA). Reverse transcription was performed using a kit purchased from Thermo Fisher Scientific, Inc., Waltham, MA, USA (Maxima^®^ First Strand cDNA Synthesis Kit) and samples were then incubated in the Advanced Biometra Product line (Analytik Jena AG, Göttingen, Germany) for 10 min at 25 °C, 15 min at 50 °C, and 5 min at 85 °C. A qRT-PCR analysis was performed with Power SYBR-Green PCR Master Mix, samples’ cDNA, sense and antisense primers, and pure water, using a Quant Studio 5 real-time PCR system. For these experiments, the following primers were used: 18S (as housekeeping genes), Bax, Bcl-2, Bad, caspase 3, and caspase 8. The comparative threshold cycle (2-Ct) method was used to calculate and express the results.

### 2.8. Combination-Index Calculation

The evaluation of the association between ASA and 5-FU was conducted using the Chou-and-Talay principle, which involves calculating several parameters, such as the inhibitory effect (Fa), the dose-reduction index (DRI), and the combination effect. CompuSyn version 1.0 software (ComboSyn, Inc., Paramus, NJ, USA) was used to analyze the potential synergistic effects. Using the combination index (CI), the combined effect can be classified as follows: synergistic effect (CI < 1), additive effect (CI = 1), and antagonistic effect (CI > 1). Additionally, by calculating the DRI parameter and obtaining a value greater than 1, combining the two compounds can reduce the dose used in monotherapy [40,41]. 

### 2.9. Statistical Analysis

The statistical analysis of the results was performed using GraphPad Prism version 9.3.1 software (GraphPad Soft-ware, San Diego, CA, USA, www.graphpad.com). The results were expressed as ± standard deviations (SD). The use of ANOVA followed by Dunett’s multiple post-test comparisons determined the statistical difference between the groups and was expressed as * (* *p* < 0.1; ** *p* < 0.01; *** *p* < 0.001; **** *p* < 0.0001).

## 3. Results

### 3.1. Cellular-Viability Assessment

The cell viabilities of the healthy human keratinocyte cell line and the colorectal adenocarcinoma tumor cell line were evaluated to investigate the pharmacotoxicological profiles of ASA, 5-FU, and the ASA + 5-FU combination.

**Figure 1:**
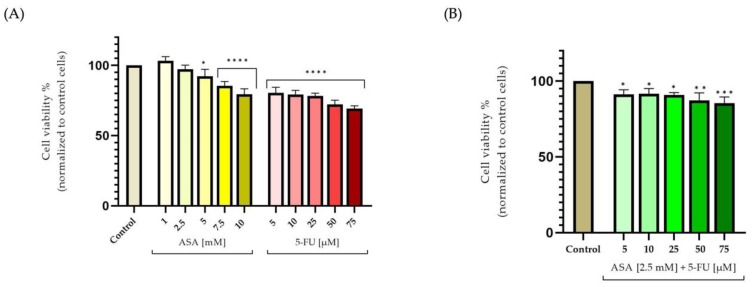
In vitro assessment of the cytotoxic effects at the level of HaCaT cells after 72 h of treatment of: (**A**) acetylsalicylic acid—ASA (1, 2.5, 5, 7.5, and 10 mM) and 5-Fluorouracil—5-FU (5, 10, 25, 50, and 75 μM); and (**B**) combination of ASA (2.5 mM) and 5-FU (5, 10, 25, 50, and 75 μM). The results are expressed as percentages, based on the standard deviation of three independent experiments. Statistical analysis was conducted using the one-way ANOVA method and Dunnett’s multiple-comparisons post-test (* *p* < 0.1; ** *p* < 0.01; *** *p* < 0.001; **** *p* < 0.0001).

The ASA was found to exhibit a dose-dependent decrease. In this case, however, the 1 mM concentration caused a slight increase in keratinocyte viability, of approximately 103%, while the 10 mM concentration resulted in a decline in the percentage of viable cells, down to about 79%. As a result of the treatment of human keratinocytes with 5-FU for 72 h, the percentage of viable cells decreased in a dose-dependent manner. Accordingly, at the lowest tested concentration (5 μM), the cell viability was approximately 80%, while at the highest tested concentration (75 μM), a percentage of 69% was obtained (Figure 1A). In contrast, the association between ASA (2.5 mM) and 5-FU (5–75 μM) led to a reduction in cytotoxicity; thus, at the highest concentration studied, the cell viability amounted to approximately 85% (Figure 1B).

**Figure 2:**
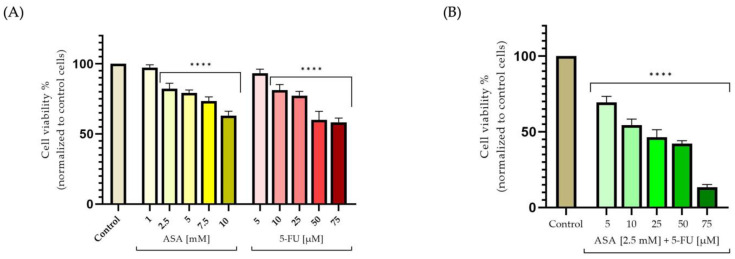
In vitro assessment of the cytotoxic effects at the level of HT-29 cells after 72 h of treatment of: (**A**) acetylsalicylic acid—ASA (1, 2.5, 5, 7.5, and 10 mM) and 5-Fluorouracil—5-FU (5, 10, 25, 50, and 75 μM); and (**B**) combination of ASA (2.5 mM) and 5-FU (5, 10, 25, 50, and 75 μM). The results are expressed as percentages, based on the standard deviation of three independent experiments. Statistical analysis was conducted using the one-way ANOVA method and Dunnett’s multiple-comparisons post-test (**** *p* < 0.0001).

Furthermore, the ASA caused a reduction in the colorectal cells’ viability; however, this was not as marked as that caused by the 5-FU. The cell viability decreased at a concentration of 5 mM (approximately 79%), but the strongest cytotoxic effect was recorded at a concentration of 10 mM, where the cell viability measured approximately 63%. Furthermore, the 5-FU caused a decrease in cell viability in the colorectal adenocarcinoma cells in close correlation with the dose tested. These results indicate that the concentration of 5 μM did not significantly decrease the cell viability (Figure 2A). By contrast, at a concentration of 10 μM, the cell viability began to decline by a value of 81%, whereas at a concentration of 75 μM, a significant drop was observed, with a value of 58%. An increase in the cytotoxic potential was observed in the HT-29 cells following the association of the two compounds. Thus, at the lowest concentration, the cell viability was significantly reduced (approximately 69%); however, at the highest concentration, the most intense cytotoxic effect was observed, resulting in a rate of viable cells of approximately 13% (Figure 2B).

### 3.2. Cellular Morphology

The impact of the tested compounds on the cell morphology after 72 h of treatment was evaluated to gain a better understanding of their cytotoxic potential.

**Figure 3:**
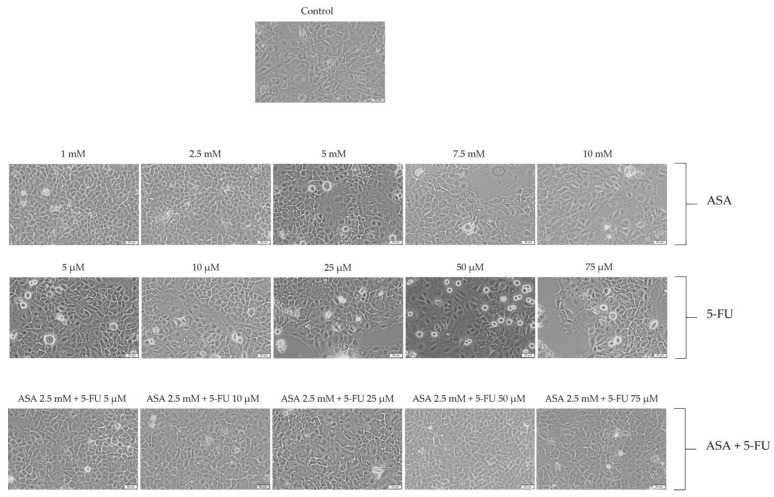
Evaluation of the effects of 72 treatments with ASA, 5-FU, and ASA+5-FU on the morphologies of HaCaT cells. Scale bars indicate 50 µm.

The ASA caused a reduction in cell number, as well as rounded and shrunken cells, especially at a concentration of 10 mM, as shown in Figure 3. Furthermore, the 5-FU treatment of the HaCaT cells caused a series of morphological changes, with the most noticeable changes occurring at concentrations of 50 and 75 μM. It was observed, in this case, that the number of cells decreased and that the cells changed their shape, becoming round and detached from the plaque, both of which are signs of cell death. As a result of the association of the two compounds, the morphological changes in the cells were not significant, exhibiting shapes and numbers similar to those of the control cells. A few morphological changes were observed, particularly at the highest concentration (ASA 2.5 mM + 5-FU 75 uM), with floating round cells observed (Figure 3). 

**Figure 4:**
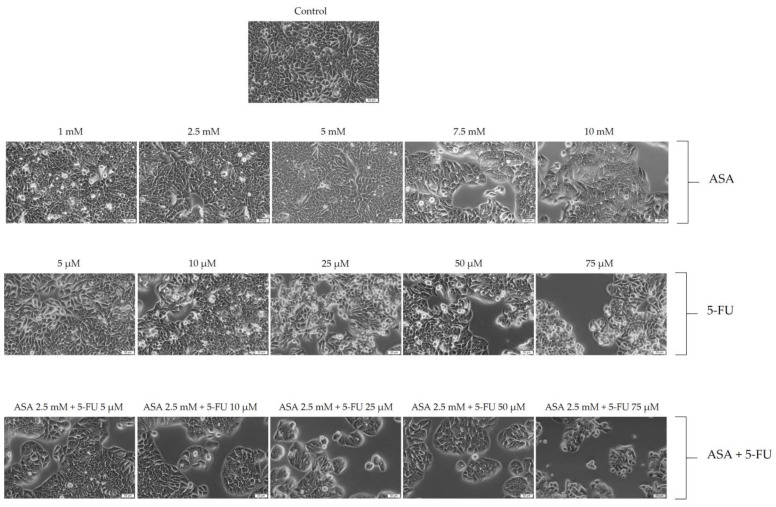
Evaluation of the effects of 72 treatments with ASA, 5-FU, and ASA+5-FU on the morphologies of HT-29 cells. Scale bars indicate 50 µm.

In the colorectal adenocarcinoma cells, the ASA induced cytotoxic effects at the level of cell morphology, manifested by a decrease in the number of cells and a change in their shape, becoming round and shrunken. A similar trend was observed with the 5-FU, but in a much more intense manner. A 72-hour stimulation with the highest concentration led to a decrease in the number of cells, with floating cells with rounded shapes observed. The most intense cytotoxic effects were, however, observed when the ASA was combined with 5-FU. An important characteristic of this case was the reduced number of cells, along with the loss of connections between the neighboring cells and the rounding of the cell shape (Figure 4). The results of these studies are consistent with those from previous analyses of cell viability. 

### 3.3. Immunofluorescence

Given the dynamic nature of the cytoskeleton, which plays a crucial role in maintaining cellular shape and morphology, the potential synergistic effects of the ASA and 5-FU were examined at the level of actin filaments. Furthermore, DAPI was used to stain the nuclei in order to gain a better understanding of the cell morphology. On the basis of the previously obtained results, the following subcytotoxic concentrations were selected: ASA 2.5 mM, 5-FU 25 μM, and ASA 2.5 mM + 5-FU 25 μM.

**Figure 5:**
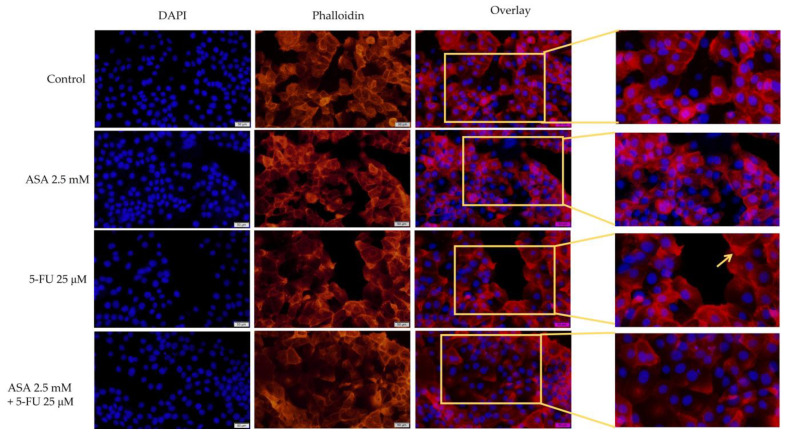
Evaluation of the effects of 72 treatments with ASA, 5-FU, and ASA+5-FU on the structures of nuclei (blue) and F-actin fibers (red) of HaCaT cells. Pictures were taken with the 20× objective, scale bars indicating 50 µm. The yellow arrows indicate the main changes (nuclear condensation, reorganization of actin filaments).

Regarding the effect on the human keratinocytes, no major differences were observed between the control cells and the treated cells. There were only small changes observed with the 5-FU, such as the condensation of chromatin and actin filaments. The nuclei and actin filaments of the cells treated with ASA and 5-FU had a uniform distribution, similar to those of the control cells (Figure 5). 

**Figure 6:**
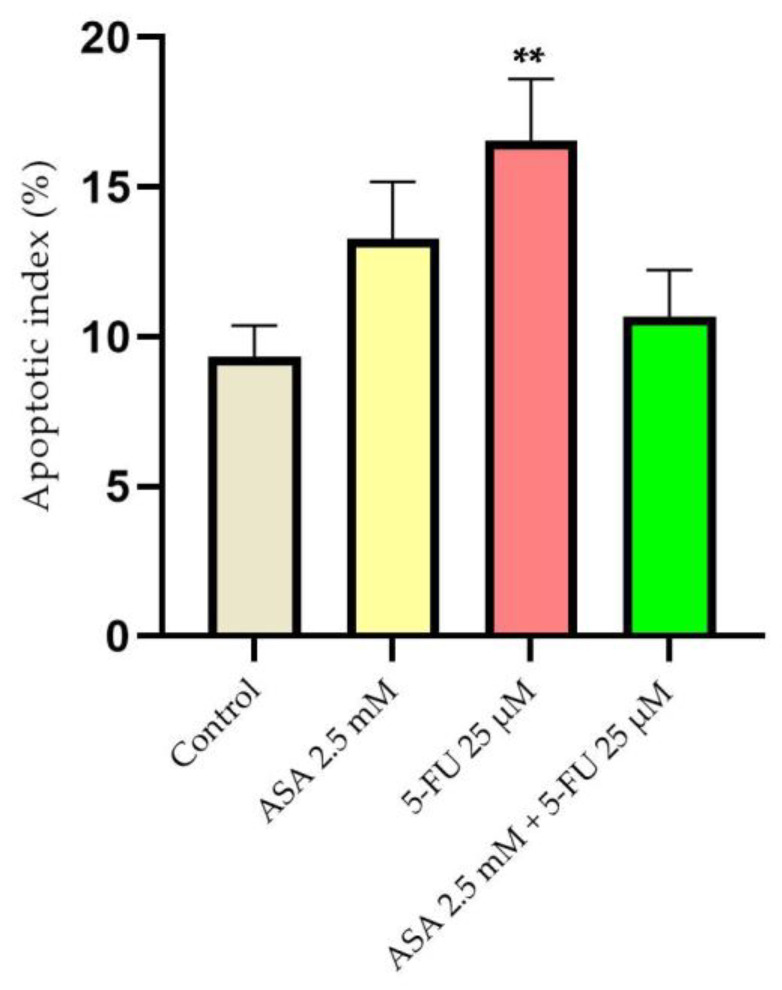
Evaluation of the apoptotic index (AI) of human keratinocytes, HaCaT, after stimulation with ASA 2.5 mM, 5-FU 25 μM, and a combination of the two compounds for a period of 72 h. The results are expressed as percentages and standard deviation as a result of three independent experiments. The statistical analysis involved one-way ANOVA analysis and a Dunnett’s multiple-comparisons post-test (** *p* < 0.01).

Based on the apoptotic index, the 5-FU induced a greater extent of apoptotic changes in the nuclei, while the ASA and 5-FU combined caused no significant changes in the nuclei (Figure 6). 

**Figure 7:**
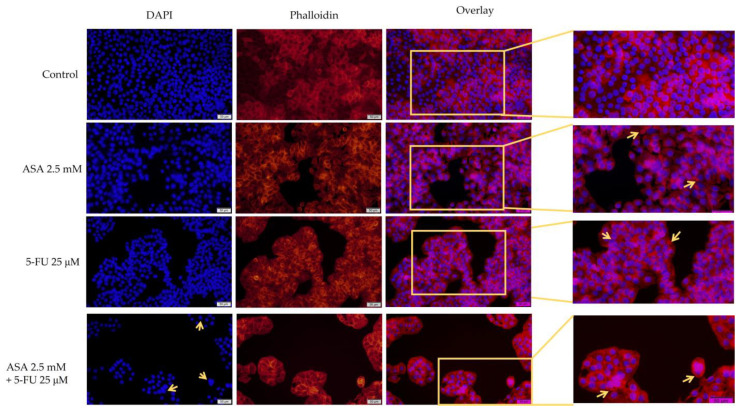
Evaluation of the effects of 72 treatments with ASA, 5-FU, and ASA+5-FU on the structures of nuclei (blue) and F-actin fibers (red) of HT-29 cells. Pictures were taken with the 20x objective, scale bars indicating 50 µm. The yellow arrows indicate the main changes (nuclear condensation, reorganization of actin filaments).

The ASA demonstrated changes at the level of the nuclei and actin filaments within the colorectal adenocarcinoma cells, which led to the appearance of chromatin condensation and cytoskeleton reorganization. In addition to reducing the number of cancerous cells, the 5-FU caused chromatin condensation and the formation of apoptotic bodies in the colorectal adenocarcinomas. Additionally, compared with the control cells, the actin filaments appeared to be reorganized and condensed. The association between the two compounds resulted in the strongest morphological changes, including the condensation and fragmentation of the chromatin, the formation of apoptotic bodies, and the reorganization of actin filaments into a condensed peripheral ring (Figure 7). It is important to note that all of these changes are indicative of apoptosis.

**Figure 8:**
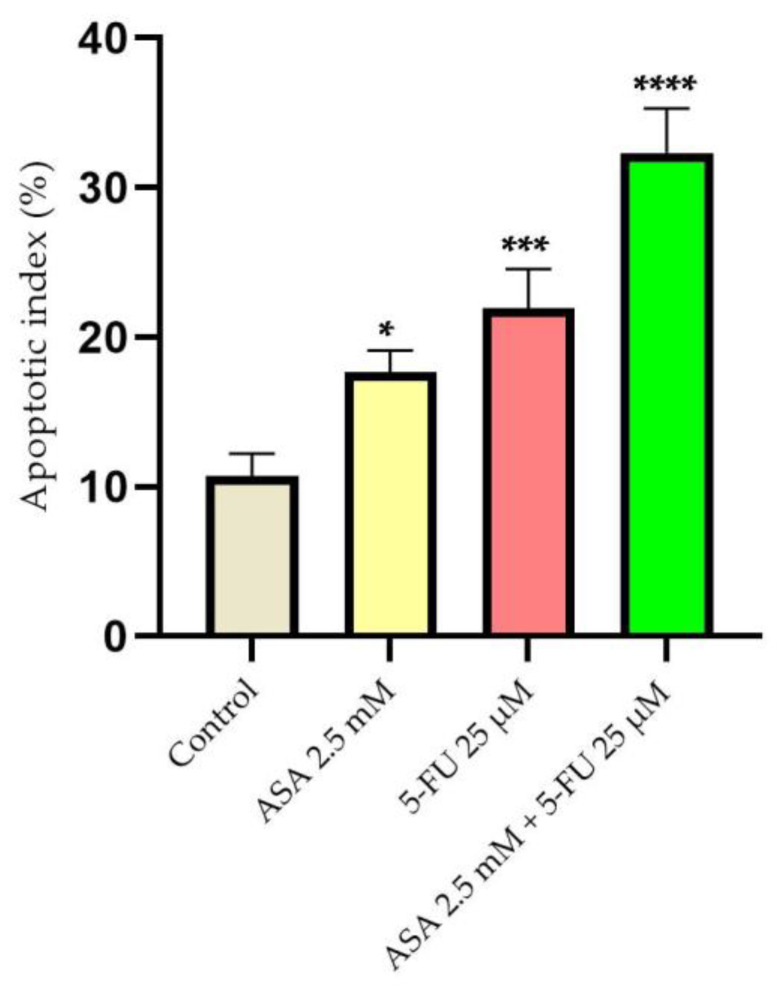
Evaluation of the apoptotic index (AI) of HT-29 cells after stimulation with ASA 2.5 mM, 5-FU 25 μM, and a combination of the two compounds for a period of 72 h. The results are expressed as percentages and standard deviation as a result of three independent experiments. The statistical analysis involved one-way ANOVA analysis and a Dunnett’s multiple-comparisons post-test (* *p* < 0.1; *** *p* < 0.001; **** *p* < 0.0001).

According to the calculation of the apoptosis index, both the ASA and the 5-FU induced changes in the nuclei of the HT-29 cells. However, the combination of the two compounds caused the most pronounced apoptotic changes (Figure 8).

### 3.4. Wound-Healing Assay

**Figure 9:**
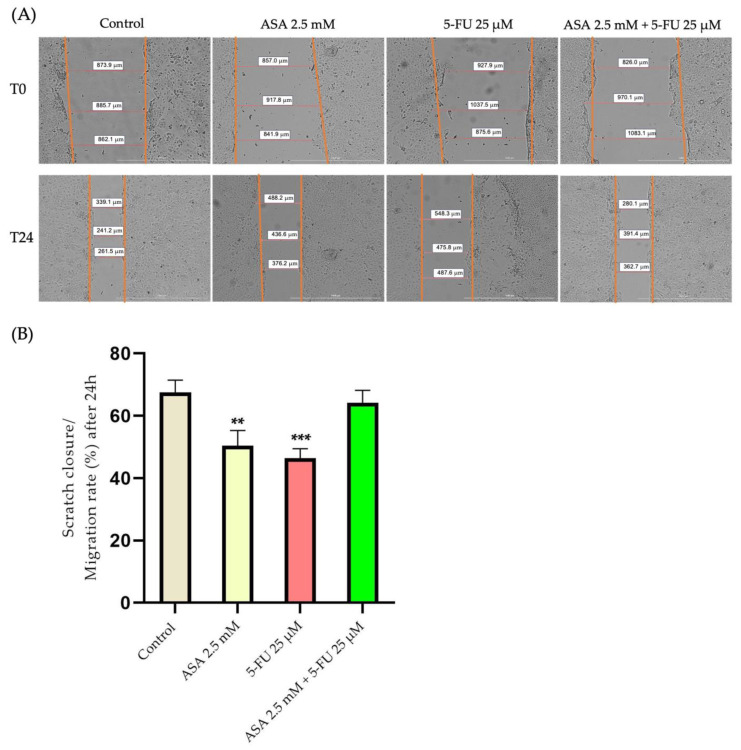
(**A**) Photographic evaluation of the migration capacity of HaCaT cells after 24 h treatment with ASA (2.5 mM), 5-FU (25 μM), and ASA 2.5 mM + 5-FU 25 μM. The images were taken at the beginning of the experiment (0 h) and at the end (24 h). The scale bars indicate 1000 µm. (**B**) Quantitative analysis of post-treatment migration capacity. The results are expressed as a percentage as the migration rate after 24 h of treatment and as the standard deviation of three independent experiments. The statistical analysis was carried out by the one-way ANOVA method followed by the Dunett’s multiple-comparisons post-test (** *p* < 0.01; *** *p* < 0.001).

To investigate the impact of the compounds on the cell migration, subcytotoxic concentrations of the compounds were selected, which did not significantly reduce cell viability, with the ASA 2.5 mM, 5-FU 25 μM, and their combination. Among the HaCaT cells, the highest migration rate was found in the control, unstimulated cells. A decrease in the migration rate of approximately 50% was recorded in the cells stimulated with ASA compared with the control cells, which had a migration rate of approximately 67%. The strongest inhibition of cell migration was observed when the cells were treated with 5-FU, where the migration rate decreased by approximately 46%. As a result of the association between the two compounds, the antimigratory effect diminished, resulting in a similar migration rate to that observed in the control cells (approximately 64%) (Figure 9).

**Figure 10:**
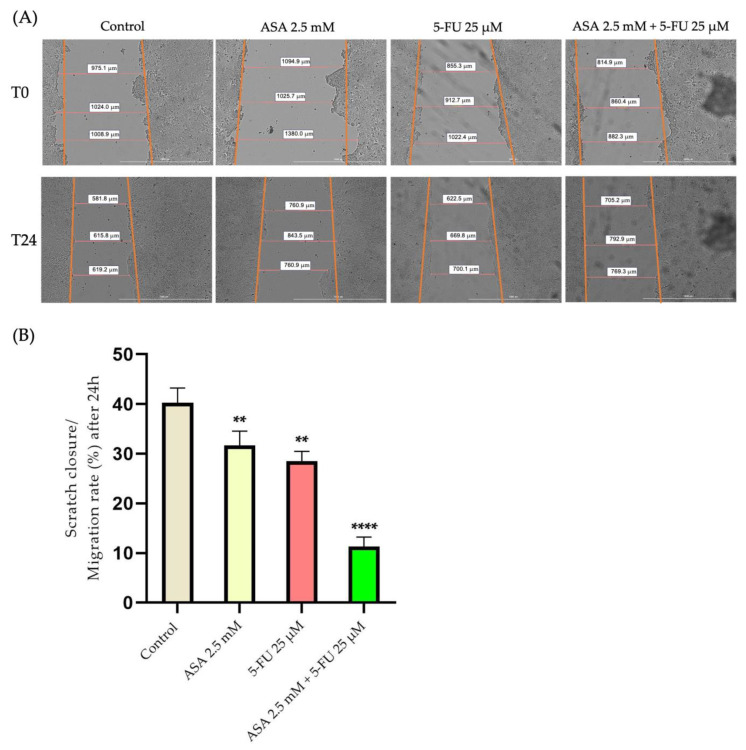
(**A**) Photographic evaluation of the migration capacity of HT-29 cells after 24 h treatment with ASA (2.5 mM), 5-FU (25 μM), and ASA 2.5 mM + 5-FU 25 μM. The images were taken at the beginning of the experiment (0 h) and at the end (24 h). The scale bars indicate 1000 µm. (**B**) Quantitative analysis of post-treatment migration capacity. The results are expressed as a percentage as the migration rate after 24 h of treatment and as the standard deviation of three independent experiments. The statistical analysis was carried out by the one-way ANOVA method followed by the Dunett’s multiple-comparisons post-test (** *p* < 0.01; **** *p* < 0.0001).

At the same time, for the HT-29 cells, the highest migration rate was recorded in the control cells. At the level of colorectal adenocarcinoma cells, the ASA determined a decrease in the migration capacity of up to 32%; in the control cells, the migration rate was approximately 40%. A stronger inhibition of cell migration was recorded with the 5-FU, which caused a decrease in the migration rate of up to 28%. Nevertheless, the greatest effect was seen when the two compounds were combined, where the migration rate was reduced by approximately 11% (Figure 10).

### 3.5. Quantification of Apoptotic Markers by Real-Time PCR

Considering the previous results, which indicated that the tested compounds, especially their combination, exhibited a marked cytotoxic effect on the HT-29 cells, causing morphological changes characteristic of cell apoptosis, the next step was to evaluate the impact on pro-apoptotic (Bax and Bad) and anti-apoptotic (Bcl-2) markers, as well as the expression levels of caspase 3 and 8. 

**Figure 11:**
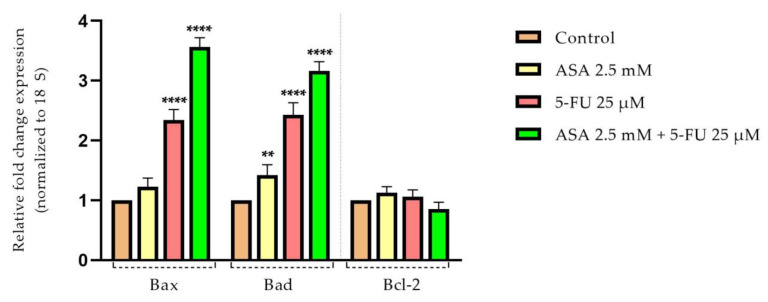
Relative fold expression of mRNA expression of pro- and anti-apoptotic mitochondrial markers in HT-29 cells after stimulation with ASA (2.5 mM), 5-FU (25 µM), and ASA 2.5 mM + 5-FU 25 μM for 72 h. The results were reported for 18 s and for the control group (non-stimulated cells) and expressed as mean values ± SD as a result of three independent experiments. The statistical analysis was performed by applying the one-way ANOVA method and Dunnett’s post-test (** *p* < 0.01, **** *p* < 0.0001).

The pro-apoptotic markers, Bax and Bad, showed an upregulation of mRNA expression after stimulation with ASA and 5-FU. The most profound effect was observed following the stimulation with both compounds combined. It should be noted that the level of mRNA expression of the anti-apoptotic marker (Bcl-2) did not change significantly after testing the compounds and remained relatively close to the control level (Figure 11). 

**Figure 12:**
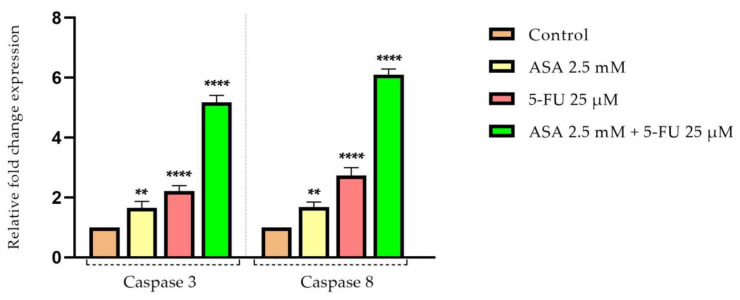
Relative fold expression of mRNA expression of caspases 3 and 8 in HT-29 cells after stimulation with ASA (2.5 mM), 5-FU (25 µM), and ASA 2.5 mM + 5-FU 25 μM for 72 h The results were reported for 18 s and for the control group (non-stimulated cells) and expressed as mean values ± SD as a result of three independent experiments. The statistical analysis was performed by applying the one-way ANOVA method and Dunnett’s post-test (** *p* < 0.01, **** *p* < 0.0001).

In addition, the caspase-3 and -8 levels were increased following stimulation with ASA or 5-FU, with the 5-FU showing the most intense effects. However, the combination of the ASA and the 5-FU had the greatest impact on the increases in the expression of caspases-3 and -8 after testing for 72 h, with both caspase types affected similarly (Figure 12).

### 3.6. Combinatio-Index Calculation

The Chou–Talalay method was applied to examine the potential synergistic effect of the association of the two compounds, with the parameters of interest calculated using CompuSyn software version 1.0.

The values of the Fa parameter, which represents the inhibitory effect, were used to calculate the dose-reduction index (DRI) and combination index (CI) parameters for the human keratinocyte cell line—HaCaT. The CI was greater than 1 at all the concentrations examined, indicating that at the level of healthy cells, the two compounds had an antagonistic effect, canceling out the cytotoxic effects. A value of greater than 1 was also observed for the DRI parameter for the 5-FU at all five concentrations tested, which indicates that the concentration of 5-FU can be reduced in a favorable manner when combined with ASA (Table 1).

To calculate the DRI and CI, the FA parameters for the colorectal adenocarcinoma cells were 0.69; 0.54; 0.46; 0.42, and 0.13. For all five concentrations tested, the CI values were less than 1, suggesting that ASA and 5-FU have synergistic effects. Additionally, the DRIs of both the ASA and the 5-FU were greater than 1, suggesting that the combined use of both compounds may lead to a favorable reduction in dose (Table 2).

## 4. Discussion

The process of the development of colorectal adenocarcinoma is characterized by the transformation of normal epithelium into a precancerous lesion, called intermediate adenomas, which leads to the development of invasive carcinoma and the formation of metastases [42]. The disease is associated with a number of risk factors, including, in addition to environmental and lifestyle factors and advanced age, the presence of comorbidities, as cardiovascular conditions [43,44,45]. Aspirin is a well-known antiplatelet agent, and it is one of the most commonly prescribed drugs for preventing secondary cardiovascular complications [46]. Several recent studies indicated that aspirin possesses antitumor properties and may offer potential therapeutic and chemopreventative benefits. To date, the most significant epidemiological results have been obtained regarding the protective and antitumor properties of ASA against colorectal cancer [47,48,49]. Systemic chemotherapy for CRC includes 5-FU as a key component. However, modern therapeutic strategies have been developed to improve antitumor activity in response to problems related to the development of resistance to treatment [50]. Oncological therapies include the combination of conventional chemotherapy with other treatments in order to improve treatment response. Nevertheless, this combined therapy entails several serious side effects [51]. Based on these premises, the present study evaluated the antitumor potential of ASA, both alone and in combination with 5-FU. 

According to the results of the viability test, ASA does not have a particularly pronounced cytotoxic effect on healthy human keratinocytes. At the highest tested concentration (10 mM), the cell viability was approximately 79%. In contrast, the 5-FU induced a greater decrease in cell viability than the ASA, with the percentage of viable cells dropping below 70%. The combined effect of these two compounds, however, reduced the cytotoxic effect on the HaCaT cells, resulting in a minimum viability of 85%. It appears that the toxic effects of the two compounds can be diminished when they are combined. Instead, when tested on colorectal adenocarcinoma cells, the compounds alone showed a moderate cytotoxic effect, decreasing the cell viability to approximately 63%, in the case of the ASA, and 58%, in the case of the 5-FU. Furthermore, when the compounds were associated, the cytotoxic effect was enhanced, with approximately 13% of the cells remaining viable. A thorough review of the literature in the field led to the selection of the concentrations of 5-FU used in this study [51,52,53]. A study conducted by Srimuangwong et al. assessed the effects of 5-FU at doses between 5 and 25 μmol/L on HT-29 cells, and it was found that the concentration of 25 μmol/L resulted in a reduction in viability below 80% after 48 h [54]. Furthermore, Zhang et al. studied the effects of 5-FU on HT-29 cells after 72 h of treatment with concentrations between 1 and 100 μmol/L. According to that study, up to 60% of the viability of the cells was reduced [55]. Additionally, Lim et al. investigated the effects of 5-FU on colorectal adenocarcinoma cells and found that they were reduced in viability in the same way as in the previous studies [56]. In this study, the human keratinocyte cell line, HaCaT, was used as a non-tumor model. These HaCaT cells are human epidermal keratinocytes that were used for the investigation of multistep carcinogenesis in human cells [57]. This cell line was chosen as a control based on its extensive use as a model for colorectal cancer research up to this point [58,59,60,61,62].

Using HaCaT cells, Rajagopalan et al. evaluated the cytotoxic potential of 5-FU (0.01–100 μg/mL). According to their findings, the cell viability was reduced by approximately 73% at the highest concentration tested [63]. These results are in accordance with the findings of the current study. The literature review provided the basis for the choice of concentrations of ASA in the present study [64,65,66]. In addition, the correlation between the dose used in vitro and the concentration used in vivo was estimated mathematically based on the formula provided by Levy [67]. Thus, the equivalent concentrations in mg of the concentrations used in vitro in this study ranged between 180 and 1800 mg/L. Accordingly, these concentrations corresponded to the range of concentrations used in vivo between 18 and 180 mg. Aspirin is generally used to prevent cardiovascular complications in vivo at a dosage of 60 to 500 mg per day [68]. In light of these considerations, the concentrations used in the present study were established around the lowest concentrations used in patients. Multiple observational studies have demonstrated the potential efficacy of non-steroidal anti-inflammatory drugs and, implicitly, aspirin in the prevention of colorectal cancer [69,70,71]. An earlier study, conducted by Ashktorab, produced similar results to those obtained in this research. Accordingly, HT-29 cells showed a reduction in viability at concentrations between 0.5 and 1.5 mM of ASA [72]. Furthermore, Ying et al. reported that ASA at 10 mmol/L decreased the proliferation and metastasis of colorectal cancer cells—HCT116 [73]. Moreover, Voutsadakis et al. pointed out that ASA was cytotoxic to HT-29 cells in concentrations and conditions similar to those in the present study [74]. The ASA was tested at concentrations ranging from 0.1 to 10 mM in human keratinocytes in the study conducted by Zhuang. According to the results of that study, ASA has no cytotoxic effect on HaCaT cells [75]. There is a strong correlation between these results and the findings of the present study.

Furthermore, 5-FU was found to cause changes in the morphologies of HT-29 cells in addition to affecting the cell viability. The highest tested concentration (75 μM) determined the rounding of the cells and their detachment from the plate. Meanwhile, the ASA influenced morphological changes to a lesser extent. It is noteworthy that the combination of ASA and 5-FU resulted in a marked decrease in the number of cells attached to the plate, a loss of connections between neighboring cells, and a reduction in their size. Additionally, at the level of the cellular organelles (nuclei and actin filaments), morphological changes were observed. For the staining of the nuclei, DAPI was selected because it is a simple staining protocol, has a high level of specificity, and is readily available for quantitative analyses [76]. The DAPI-staining protocol can be used on live or fixed cells, with a fluorescence that is 20 times more pronounced when bound to DNA [77]. Additionally, phalloidin staining was used to visualize the actin filaments. By using fluorescent phalloidin, actin filaments are stabilized and can be visualized. It is well known that rhodamine–phalloidin exhibits a much higher fluorescence when bound to actin filaments [78]. The most pronounced changes were observed with the combination of ASA and 5-FU. Among these changes were the condensation of the nucleus, the decrease in nucleus size, the formation of apoptotic bodies, and the reorganization of the cytoskeleton, with actin filaments organized into a peripheral ring. All of these changes were indicative of an apoptosis-like effect. Conversely, only the 5-FU resulted in morphological changes characteristic of cell death in the HaCaT cells. As a process of cell death, apoptosis is characterized by a series of changes occurring both within the nucleus and within the cytoplasm [79]. At the level of the nucleus, apoptosis is associated with changes in the organization of chromatin, the development of crescent shapes at the nuclear periphery, the condensation of chromatin, the formation of apoptosis bodies, the contraction of the nucleus, and a reduction in the size of the nucleus [80]. In addition to the changes that occur within the nucleus, apoptotic cells also undergo a reorganization of their cytoskeleton, which results in a decrease in volume and contraction. An immunocytochemical analysis revealed that actin filaments reorganize during the apoptotic process into bundles that become increasingly thick and are located mainly on the periphery of cells [79]. Because the actin cytoskeleton is a key component of most cellular functions, its spatial and temporal dynamics may change rapidly in response to various physiological or pathological conditions. As a result of the apoptosis process, the actin cytoskeleton undergoes reorganization into a peripheral actomyosin ring. In addition, actin machinery proteins are important substrates for caspases; therefore, actin is involved in the initiation and execution of apoptosis [81]. For the visualization of actin filaments, phalloidin and its fluorescent derivatives are considered the gold standards. The advantages of the use of phalloidin and its derivatives are numerous and include: (i) the fact that it is a convenient method for intensifying, quantifying, and labeling the formation of F-actin filaments in cells; (ii) the high and selective affinity of phalloidin for F-actin, reducing non-specific staining and background noise; and (iii) the provision of the finer details of actin filaments due to specific staining [82].

The changes previously described were mostly observed in HT-29 cells treated with ASA+5-FU. Choi et al. investigated the effect of aspirin on the nuclei of breast cancer cells—MCF-7. After 24 h of treatment with ASA at a concentration of 5 mM, the researchers reported that ASA induces strong condensation at the chromatin level [83]. In addition, the effect of ASA on the nucleus was studied in human megakaryoblast cells. Therefore, in the research conducted by Massimi et al., DAPI staining revealed that ASA induces strong condensation of the nucleus [84]. Moreover, Bashir et al. pointed out that ASA provokes changes characteristic of the apoptotic process at the nucleus level of colorectal cancer cells. It was shown that ASA at a concentration of 1 mM initiates chromatin condensation, a change that was also observed in the present study [85]. A study conducted by Saber et al. examined the effects of 5-FU at the nuclei level and concluded that treatment with 5-FU at a concentration of 7 μg/μL results in a strong condensation of the nuclei [86]. Furthermore, 5-FU caused a reorganization of actin filaments within smooth muscle cells at a concentration of 10 mM, resulting in the condensation of actin filaments. Moreover, there was a reduction in the number of actin filaments compared to the control group. These results explain the adverse reactions that occurred following treatment with 5-FU at the level of gastrointestinal motility [87]. It should be noted that in the above study, the concentration of 5-FU was more than 100 times higher than that tested in the present study. 

Furthermore, the actin cytoskeleton contributes significantly to cell migration [88]. A tumor’s ability to invade and metastasize is also dependent upon its ability to migrate [89]. Based on these considerations and taking into account that the actin filaments of the tumor cells displayed significant changes, the next phase of the study was to determine the migration capacity of the cells after treatment with ASA, 5-FU, and the combination of both. According to the scratch method, ASA and 5-FU inhibit the migration of human keratinocytes, but their association did not cause a significant inhibition compared to the control. However, both compounds inhibited cell migration at the level of colorectal adenocarcinoma cells, while their combination had a much more intense antimigratory effect than either alone. Cell migration can be evaluated using scratch assays, which are among the easiest and fastest methods available. This test evaluates the ability of whole cell masses to migrate and allows the observation of cell morphology during migration [90]. According to a study published by Yokomizo et al., aspirin has a negative impact on skin-wound healing by reducing 12-hydroxyheptadecatrienoic acid, thus inhibiting keratinocyte migration [91]. The ASA, however, was shown to be effective in inhibiting cell migration at the level of several types of tumor cell. Consequently, Ma et al. investigated the antimigratory effects of aspirin (2–8 mmol/L) on HT-29 cells, finding that aspirin inhibited the migration of the cells [65]. The scratch method was also used in a study by Ying et al. to demonstrate that the aspirin treatment of colorectal cancer cells (HCT-116 and C26) inhibits migration by reducing the expression of toll-like receptor 4 and the epithelial–mesenchymal-transition phenotype [73]. In recent studies, it has been demonstrated that COX-2 is involved in the migration of human colon cancer cells through the activation of the epithelial–mesenchymal transition. This consideration may provide one explanation for the antimigratory effects of aspirin [92]. Regarding 5-FU, it is known that, as with other traditional chemotherapy agents, it does not possess a selective cytotoxic effect. Consequently, treatment with 5-FU is associated with several side effects, including dermatitis, caused by 5-FU’s damaging effects on keratinocytes [93]. At the same time, 5-FU may also have an antimigratory effect on tumor cells. Accordingly, Yu et al. examined the effect of 5-FU on the migration of colorectal cancer cells and found that 5-FU had anti-migratory properties similar to the results reported in the present study [94]. Although 5-FU exhibits an antimigratory effect on colorectal cancer cells, its mechanism of action is not completely understood. Based on the study conducted by Wang et al., 5-FU inhibits the epithelial–mesenchymal transition in colorectal adenocarcinoma cells [95]. In addition, Seo et al. demonstrated that 5-fluorouracil inhibits colon-cancer-cell migration through the activation of the antioxidant enzyme, sestrin 2 [96]. 

The process of apoptosis is an extremely important biological process that contributes both to tumor development and to the reduction in effectiveness of conventional antitumor therapy. The B-cell lymphoma 2 (Bcl-2) family of proteins includes members that possess both antitumor and protumor properties. There has been significant interest in Bcl-2 proteins in recent years due to their crucial role in the regulation of apoptosis, tumorigenesis, and therapeutic response [97]. Cell death or survival is determined by the interaction between three fractions of the Bcl-2 family, namely: (i) anti-apoptotic, survival factors (e.g., Bcl-2); (ii) pro-apoptotic factors, BH3 proteins (e.g., Bad); and (iii) effectors of cell death (e.g., Bax) [98]. Therefore, both ASA and 5-FU, as well as their combination, were assessed to determine their influence on the expression of three genes involved in the apoptosis process: Bax, Bad, and Bcl-2. Compared to the control, the combination of the two compounds increased the expression of proapoptotic markers, while Bcl-2 expression was relatively reduced. As reported by Zhou et al., ASA is associated with a decrease in Bcl-2 expression and an increase in Bax and Bad expression in gastric cancer cells [99]. Choi et al. also reported that ASA reduces Bcl-2 expression in breast cancer cells, which is associated with increased nuclear chromatin condensation [83]. In accordance with the previous studies, reducing the expression of Bax facilitates colorectal cancer development and increases the resistance of tumor cells to 5-FU treatment. As a consequence of increases in Bax expression, tumor cells become more sensitive to treatment, which enhances 5-FU’s therapeutic efficacy [19,100,101,102]. According to early studies, 5-FU exerts its cytotoxic effect on colorectal cancer cells by modulating Bcl-2 family proteins [103]. As part of the process of apoptosis, apoptotic proteases, also known as caspases, are activated, which selectively cleave cell substrates [104]. As a result of activating the members of the pro-apoptotic Bcl-2 family (Bax and Bak), cytochrome c is released, which contributes to caspase-9 activation through its binding to Apaf-1. Upon activation, caspase 9 cleaves and activates caspase 3. Known as the executioner caspase, caspase 3 contributes to the alteration of the structure of cells by fragmenting DNA and degrading proteins in the cytoskeleton [105]. 

Moreover, caspase-8 is considered the initiator caspase of extrinsic apoptosis [106]. The findings of this study indicate that the association of 5-FU and ASA leads to an increase in the expression of both caspases, caspase 3 and caspase 8, in colorectal adenocarcinoma cells. Zimmermann et al. reported in an initial study that aspirin induces apoptosis in cervical cancer cells by releasing cytochrome C from mitochondria and enhancing the expression of caspase 3 [107]. Furthermore, at a concentration of 8 mM, aspirin activated caspase 3 in human oral squamous carcinoma cells, leading to an apoptotic effect [108]. Furthermore, 5-FU has been shown to be a pro-apoptotic agent in colorectal cancer cells by activating caspase 9, which contributes to caspase-3 activation [109]. 

Previous studies explored the possible signaling pathways that ASA might interfere with, thereby exerting its antitumor effects. There are several signaling pathways through which ASA has an impact, including COX enzymes, cyclin-dependent kinases, nuclear factor (NF-)-kB, the Wnt/β-catenin pathway, the adenosine-monophosphate-activated protein-kinase pathway, the p53 acetylation pathway, and the induction of the production of p21 and Bax proteins [31]. Furthermore, ASA has been found to act preferentially on CRC cells, but the mechanism through which this action occurs has not yet been fully understood [110]. Another question that remains unanswered is how both low and high concentrations of aspirin are able to inhibit tumor growth in the colorectal region. The most plausible explanation is that ASA directly acts on the cell targets of CRC, while other mechanisms, such as COX inhibition, can contribute to the prevention of metastases [111]. In terms of its mechanism of action, 5-FU is mainly responsible for the capture of DNA and RNA, in addition to inhibiting thymidine synthase [112]. The main challenge in treating CRC with 5-FU is the development of resistance to the treatment. There is a close relationship between resistance to 5-FU and the activation of the NF-kB factor, which contributes to the increase in the expression of anti-apoptotic proteins (e.g., Bcl-2). Therefore, in vitro studies showed that cells resistant to 5-FU exhibited high levels of Bcl-2 or Bax. In this regard, along with the association with other substances that increase the expression of pro-apoptotic proteins (Bax, Bak, etc.), there is a simultaneous decrease in anti-apoptotic genes, thus increasing the sensitivity of cells to 5-FU treatment [113]. 

It is noteworthy that these results are consistent with those obtained in the present study, with respect to both the concentrations tested and the biological effects observed.

## 5. Conclusions

Finally, the results of the present study highlighted synergistic cytotoxic effects when 5-fluorouracil and aspirin were combined to target colorectal adenocarcinoma cells, HT-29. Specifically, this combination inhibits cell proliferation, alters the morphology, nucleus and cytoskeleton of cells, inhibits cell migration, and exhibits characteristic signs of cell apoptosis (decreased expression of Bcl-2 and increased expression of Bax, Bax, and caspases 3 and 8). It is important to point out that these changes were observed exclusively in tumor cells, with healthy cells not affected. Further research is necessary to fully define the profile of safety and to elucidate the biological mechanisms of action.

## Figures and Tables

**Table 1:** The parameters of the synergistic effect determined for the combination of ASA+5-FU at the level of HaCaT cells (inhibitory effect (Fa); combination index (CI) and dose-reduction index (DRI) for ASA and 5-FU).

Inhibitory Effect (Fa)	Combination Index (CI)	Dose ASA	Dose 5-FU	Dose-Reduction Index (DRI) ASA	Dose-Reduction Index (DRI) 5-FU
0.92	199.548	2.5 mM	5 μM	0.01	1.90
0.91	110.082	2.5 mM	10 μM	0.009	2.07
0.91	274.483	2.5 mM	25 μM	0.003	2.07
0.87	79.970	2.5 mM	50 μM	0.012	2.77
0.85	55.189	2.5 mM	75 μM	0.018	3.11

**Table 2:** The parameters of the synergistic effect determined for the combination of ASA+5-FU at the level of HT-29 cells (inhibitory effect (Fa); combination index (CI) and dose-reduction index (DRI) for ASA and 5-FU).

Inhibitory Effect (Fa)	Combination Index (CI)	Dose ASA	Dose 5-FU	Dose-Reduction Index (DRI) ASA	Dose-Reduction Index (DRI) 5-FU
0.69	0.46	2.5 mM	5 μM	3.044	7.094
0.54	0.31	2.5 mM	10 μM	5.276	7.937
0.46	0.35	2.5 mM	25 μM	6.951	4.754
0.42	0.46	2.5 mM	50 μM	7.993	2.916
0.13	0.10	2.5 mM	75 μM	31.045	14.178

## Data Availability

The data presented in this study are available on request from the corresponding author.
